# Utilization of peptide phage display to investigate hotspots on IL-17A and what it means for drug discovery

**DOI:** 10.1371/journal.pone.0190850

**Published:** 2018-01-12

**Authors:** Joey P. Ting, Frances Tung, Stephen Antonysamy, Stephen Wasserman, Spencer B. Jones, Feiyu F. Zhang, Alfonso Espada, Howard Broughton, Michael J. Chalmers, Michael E. Woodman, Holly A. Bina, Jeffrey A. Dodge, Jordi Benach, Aiping Zhang, Christopher Groshong, Danalyn Manglicmot, Marijane Russell, Sepideh Afshar

**Affiliations:** 1 Department of protein Engineering, Eli Lilly Biotechnology Center, San Diego, California, United States of America; 2 Department of structural Biology, Discovery Chemistry Research and Technologies, Lilly Biotechnology Center, Eli Lilly and Company, San Diego, California, United States of America; 3 Department of structural Biology, Discovery Chemistry Research and Technologies, Eli Lilly and Company, Advanced Photon Source, Argonne, Illinois, United States of America; 4 Lilly Research Laboratories, Indianapolis, Indiana, United States of America; 5 Centro de Investigación Lilly, Alcobendas, Spain; NCI at Frederick, UNITED STATES

## Abstract

To date, IL-17A antibodies remain the only therapeutic approach to correct the abnormal activation of the IL-17A/IL-17R signaling complex. Why is it that despite the remarkable success of IL-17 antibodies, there is no small molecule antagonist of IL-17A in the clinic? Here we offer a unique approach to address this question. In order to understand the interaction of IL-17A with its receptor, we combined peptide discovery using phage display with HDX, crystallography, and functional assays to map and characterize hot regions that contribute to most of the energetics of the IL-17A/IL-17R interaction. These functional maps are proposed to serve as a guide to aid in the development of small molecules that bind to IL-17A and block its interaction with IL-17RA.

## Introduction

Interleukin-17A (IL-17A) is a pro-inflammatory cytokine that plays a key role in host defense and inflammation. IL-17A is produced by activated CD4^+^ T-cells called Th17, as well as by natural killer cells, CD8^+^ NK cells, δγ T-cells, macrophages, dendritic, myeloid, and type 3-innate lymphoid cells [1–4]. Binding of IL-17A to a heteromeric receptor complex stimulates various signal transduction pathways such as NF-κb and AP-1 [5]. This triggers up-regulation of matrix metalloproteinases and various pro-inflammatory cytokines and chemokines including IL-1, IL-6, GM-CSF, CXCL-1, CCL2, and CCL7. As a result, immune cells, including neutrophils and monocytes, are attracted to the inflammation site. Elevated levels of IL-17A and the resulting cytokine release are linked to many autoimmune related diseases, including psoriasis, asthma, and rheumatoid arthritis [1–4, 6].

IL-17A protein is characterized by two pairs of β-strands that are stabilized by two disulfide bonds. The homodimeric IL-17A contains 155 amino acids and is secreted as a glycoprotein with a molecular mass of 35 KDa [5]. IL-17A belongs to a cytokine family comprised of six members. The members of this family, named IL-17A through F, share 16% to 50% amino acid identity with IL-17A, have five conserved cysteine residues, and form a similar structure. IL-17A and -F share the highest degree of homology and are found as both homo- and heterodimers. These cytokines IL-17A, IL-17F, and IL-17AF bind to common receptors IL-17RA and IL-17RC, albeit with different affinities. Whereas expression of IL-17A is restricted to immune cells, its receptors are ubiquitously expressed [5, 6].

Augmented expression of IL-17A is linked with various autoimmune disorders. Therefore, blocking activity of IL-17A has been of great therapeutic interest [7]. Two approved antibody based therapeutics targeting IL-17A, Secukinumab and Ixekizumab, are currently being used for treatment of moderate to severe psoriasis. During clinical trials, both antibodies reduced the severity of the disease by at least 75% (PASI75) in 80% of the patient population. Moreover, current advanced clinical trials have shown promising results for treatment of ankylosing spondylitis and psoriatic arthritis [7–9].

Clinical success in antagonizing IL-17A function has spurred the development of additional therapeutic modalities including orally available small molecules. Although oral drugs can provide a more convenient therapeutic option for patients; inhibition of cytokines and their receptors has thus far remained the domain of antibodies. The primary reason for this therapeutic monopoly is that cytokines, among them IL-17A, belong to a broader family of proteins that constitute protein-protein interaction (PPI) targets that have been deemed to be largely undruggable as recently as a few decades ago [10]. One reason PPIs have been challenging targets is that their interfaces are generally flat and lack deep subpockets and grooves that are usually necessary for binding of traditional organic small molecules [11]. The interaction of IL-17A with IL-17RA spans across a large surface area of approximately 2,200 Å^2^ on IL-17A [12]. The extensive nature of this interaction involves discontinuous and tertiary structural epitopes that includes both the ligand and its receptor, making the discovery of small molecule inhibitors particularly difficult. Despite these challenges, in 2013, Ensemble Therapeutics announced discovery of small molecule IL-17A antagonists [13]. It is not known whether Ensemble compounds have entered clinical trials.

Understanding the interaction of IL-17A with its receptor is a vital step for a successful drug discovery program. Interaction of IL-17A with IL-17RA–like all the other PPIs–is driven by the presence of hotspots: isolated residues that contribute a large fraction of the binding energy. Interestingly, research into PPIs has shown that hotspots are not randomly dispersed across the protein interface; rather, they form in clusters to generate a network of interactions [11, 14, 15]. Traditionally, alanine scans are used to map the functional hotspots on PPIs [16]. However, this approach is time consuming, costly, and not suited for high-throughput PPI analysis. In this paper, we have utilized a combinatorial approach using phage display to investigate the organization of hotspots on IL-17A. This has enabled us to identify disparate sites that are involved in the interaction of IL-17A with IL-17RA. Furthermore, discovery of these peptides has provided insights into the functional binding sites on IL-17A that might be amenable for small molecule binding.

## Materials and methods

### Reagents

HEPES 1 M solution was obtained from Hyclone (A Thermo Fisher Scientific Company. Waltham, MA). 10% Tween surfactant was provided by Thermo Fisher Scientific. (Waltham, MA). Sodium chloride was ordered from AccuGene (A Lonza company. Basel Switzerland). Sigma was the supplier for the human serum albumin (HSA) (Saint Louis, MO). The Dimethyl Sulfoxide (DMSO) was supplied by Acros (Thermo Fisher Scientific Company. Geel Belgium). IL-17A and IL-17RA were provided in-house by Eli Lilly and Co. (San Diego, CA)

### Expression and purification

Several different constructs of IL-17A (Reference sequence NP_002181) were prepared for structural, biophysical and biochemical studies. For HDX and ITC we prepared a construct of IL-17A residues 24–155 with a C-terminal His-tag. For crystallography, we utilized a construct of IL-17A made with residues 38–155 with the mutations N68D/C129S and a cleavable N-terminal His-tag. For TDF we used a construct of IL-17A residues 34–155 (N68D/C129S) and a C-terminal His-tag. For Surface Plasmon Resonance we designed an IL-17A molecule with a single biotin tag per dimer, by co-expressing two IL-17A constructs (one with a His-tag, and the other with Avi- and Flag- tags), together with BirA for in vivo biotinylation. IL-17RA (NP_055154) residues 1–317 with mutations N206D/N265D was expressed with a C-terminal Flag-tag.

All the constructs were expressed in insect cells. IL-17A and IL-17RA were PCR amplified and TOPO cloned into a custom TOPO adapted pFastBac (KX) vector (Thermo Fisher Scientific. Carlsbad, CA) or an off the shelf Bac to Bac HBM TOPO vector (KH) (Cat # A11338 Thermo Fisher Scientific. Carlsbad, CA). Standard baculovirus expression using a modified version of the Bac to Bac system protocol (Thermo Fisher Scientific. Carlsbad, CA) in combination with the DH10EMBacY bacmid (Geneva Boiotech. Geneva, Switzerland) was used to generate virus. Fermentations of IL-17 and IL-17RA in *Sf*9 cells were 72 hrs in length, harvested by centrifugation and supernatants were transferred for purification. The different constructs of IL-17A and IL-17RA were purified from baculovirus by Flag and/or Nickel affinity chromatography followed by size exclusion chromatography. Numbering used in IL-17A constructs refer to the gene sequence, therefore the precursor protein.

### Phage display library construction and selection

Peptide phage libraries were generated using the previously described IX104 bacteriophage vector [17]. *Escherichia coli* strain RZ1032 (ATCC 39737), which lacks functional dUTPase and uracil glycosylase, was used to prepare uracil containing single-stranded DNA (du-ssDNA) of the IX104 vector. A library oligonucleotide, containing the random amino acid sequences encoded by NNK was designed such that the random NNK region was flanked by nucleotides complementary to the vector. 5’-phosphorylated reverse complement oligo was annealed to dU-ssDNA IX104 vector using Kunkel mutagenesis and extended to form double stranded DNA (dsDNA) [18]. Electrocompetent *E*. *coli* DH10B cells (Invitrogen cat# 18290015) were used for transformations. A pool of transformants was titered to determine the diversity of each library. Phage were then amplified in the presence of freshly grown XL-1blue cells overnight on LB plates at 37°C. The next day, phage was eluted off the plate, precipitated, titered and stored at -80°C in the presence of 50% glycerol until use. For primary selection, 10^12^ phage from each library were incubated for one hour with 100 nM of Avi-tagged IL-17 at room temperature to allow binding of the peptide to the antigen. This was followed by pulling down the IL-17: phage complex using streptavidin coated magnetic beads. After washing the beads with PBS containing 0.1% Tween and PBS only, the bound phage was eluted using 100 mM of Triethylamine and was immediately neutralized with Tris/HCl pH 6.8. Phage were then amplified for three hours using mid-log XL-1blue cells, precipitated, and titered for use in subsequent rounds of selection. After three rounds of selection were completed, phage was screened to identify specific binders using filter-lift, single point ELISA (SPE), and titer dependent ELISA as described previously (methods in Molecular Biology, vol. 207). For filter-lift nitrocellulose filters were coated with 2 μg/ml of anti-M13 antibody prior to phage lift. Specific binders were identified by matching the positive response on the filter to the original plate. Positive hits were sequenced and their specific binding was confirmed by SPE and titer-dependent ELISA in the presence of 50 nM Avi-tagged IL-17. For the purpose of affinity maturation of the peptide hits secondary libraries were constructed using a monovalent display vector. Affinity maturation selection involved stringent wash conditions and an off-rate screening as described previously [19]. Primary hits were screened against 2 nM of Avi-tagged IL-17 to identify strong binders using filter-lift, SPE, and titer dependent ELISA as described above.

### Surface plasmon resonance (SPR)

BIAcore T200 (GE Healthcare) was used to determine binding affinity and kinetics of the peptides to IL-17A. All SPR experiments were performed at 8°C with flow rate of 50 μl/min. Avi-tagged IL-17A (6000–9000 RU) was captured to streptavidin sensor chip (GE Healthcare, Series S Sensor Chip SA, BR-1005-31). All peptides were reconstituted in DMSO as 10 mM stocks; then further diluted to final assay concentration series in 10 mM HEPES pH 7.5 containing 150 mM NaCl, 0.005% P20, and 3% DMSO. All peptides were tested as concentration series with step increments of 3 folds. The highest dosing concentration for each peptide was adjusted in an effort to reach binding saturation. However, saturating concentrations were not possible for some peptides. This is possibly due to binding irregularity at those concentrations induced by the peptide solubility limitation. For each SPR run, the sensor was first conditioned by 8 cycles of blank (buffer) prior to sample injections. Each SPR cycle consisted of 30–90 seconds of sample injection. This was followed by 60–300 seconds of buffer flow to monitor dissociation. Length of association and dissociation time was tailored to the kinetics of the test subjects. Sensorgrams from the IL-17A sensor were double referenced by subtracting the sensorgrams from the reference flow cell, then nearest blank. The resulting sensorgrams of the concentration series of a same peptide were fitted globally to extract binding kinetics and affinity with BIAcore T200 Evaluation Software 2.0 (GE Healthcare) using a 1:1 binding model.

### HDX-MS

HDX-MS experiments were performed as described elsewhere [20, 21]. For each experiment IL-17 was pre-incubated with peptide at a 1:2.5 molar ratio in HEPES buffer pH 7.5 prior to deuterium labeling. The on-exchange reaction was initiated by a 5-fold dilution of a 10 μM protein stock solution (in the presence or absence of peptide) in the corresponding D_2_O buffer at 4°C. The following on-exchange times were measured for each sample: 10 s, 30 s, 60 s, 300 s, 900 s and 3600 s. Each on-exchange time point was measured using three independent experiments. In addition to the on-exchange data, DMin (H_2_O only in the presence or absence of peptide) and DMax samples were measured for both samples in duplicate [22]. At each time point, HDX reactions were quenched using 3 M urea containing 500 mM of TCEP pH 4, followed by online pepsin digestion and rapid desalting through a C_8_ trap column. The resulting proteolytic peptide mixtures were separated by reversed phase liquid chromatography on an analytical C_18_ column and eluted into the mass spectrometer (Q-Exactive, Thermo Scientific). HDX-MS data were processed with HDX Workbench software [23]

### Crystallization and data collection

The IL-17A complex with peptide was prepared by incubating IL-17A at 7.1 mg/ml in 10mM Bis-Tris pH 6.5, 10% glycerol, 150mM NaCl with 2 mM of peptide. The complex was screened in sparse matrix crystallization screens at 8°C. Crystals were obtained in a sitting drop equilibrated against a reservoir containing 20% PEG 3350 and 200mM Lithium Chloride. Crystals were transferred to a reservoir solution supplemented with 20% ethylene glycol and flash frozen in liquid nitrogen for data collection. Data were collected at the Lilly Research Laboratories Collaborative Access Team (LRL-CAT) beam line at the Advanced Photon Source, Argonne, IL. Crystals belonged to the P2_1_2_1_2 Space Group with unit cell parameters a = 36.77, b = 55.12, c = 143.95 Å. The structure was determined by Molecular Replacement using the program Phaser [24] and an internal structure of IL-17A as the search model. The model was rebuilt using COOT [25] and refined using Refmac [26]. Data and Refinement statistics are included in S2 Table. The structure has been deposited with the Research Collaboratory for Structural Bioinformatics Protein Data Bank (PDB ID code 5VB9).

### IL-17A:IL-17RA AlphaLISA

The ability of compounds to inhibit the binding of IL-17A to IL-17RA was followed with an AlphaLISA assay. 0.1 μl of compounds in 100% DMSO were serial diluted using Echo Acoustic Liquid Handler (LabCyte Sunnydale, CA) into 384 well proxiplates (Perkin Elmer). A 5 ul addition of IL-17RA-flag was added to the plate, followed by the addition of 5 ul Avi-tagged IL-17A-His to provide a final concentration of 2 nM of IL-17RA and IL-17A, respectively. The assay was carried out in 20mM HEPES, 150mM NaCl, 0.05% Tween-20, 0.1%Human Serum Albumin, pH 7and 1% DMSO. The assay mixture was allowed to incubate at room temperature for 2hours. After the 2 hour incubation 10uL of streptavidin donor beads (20ug/ml final) and Anti-FLAG acceptor beads (20 ug/ml final) were added to the plate. The plate was allowed to incubate for one hour at room temp, then read on the Envision (Perkin Elmer). The relative IC50 values were determined using a four parameter fit as shown in Eq 1. In this equation, bottom and top are defined as the plateaus of the curve, and H is the Hill Slope.

No inhibitory effect was observed from addition of 0.1% DMSO only.

$$Y=Y_{bottom}+\left(Y_{top}–Y_{bottom}/1+10^{\left(log\;IC50‑x\right)*H)}\right)$$ Eq 1

### Cell-based neutralization assay

HT-29 cells (#HTB-38; ATCC) were plated in a 96 well plate (#3596; Costar) at 20,000 cells per well in McCoy’s 5A (Modified) medium (#16600–082; Gibco) supplemented with 10% FBS. Cells were treated with 60 ng/mL (1,875 pM) human IL-17A (#317-ILB; R&D Systems) in the presence of peptide at the indicated concentrations. A dose range of 0.05 nM to 50,000 nM was evaluated. After ~48 hours, CXCL1/GROα in the culture media was measured using a commercial ELISA kit (#DY275; R&D Systems). Medium alone treatments were included in every experiment to assess the basal levels of CXCL1/GROα. Percent inhibition was calculated by subtracting the mean IL-17A alone CXCL1/GROα values and then dividing by the IL-17A alone value (subtracted from the basal CXCL1/GROα level). So the differences observed at the bottom end of the curve are truly peptide-dependent.

### ELISA-based binding assay

IL-17RA-Fc, IL-17RC-Fc, IL-17RE-Fc, and IL-17RB-Fc (#177-IR, #1207-BR, #9284-IL, #358-MR; R&D Systems) were plated in 96 well plates (#655081; Greiner) at 2ug/mL in PBS for IL-17RA and 1ug/mL for all other receptors. Plates were incubated O/N at 4°C. Plates were washed 3X with TBST wash buffer (#T9039; Sigma) and blocked for 1hr with 200uL of protein-free T20 blocking buffer (#3753; Pierce). Cytokines were biotinylated with EZ Link NHS-Biotin according to manufacturer’s instructions (#21336; Thermo). Biotinylated cytokines, 250ng/mL human IL-17A (#317-ILB; R&D Systems), 100ng/mL human IL-17C (#1234-IL; R&D Systems), 100ng/mL human IL-17E (#1258-IL; R&D Systems), and 100ng/mL human IL-17F (#1335-IL; R&D Systems) were incubated in the presence of inhibitor compounds in wash buffer at the indicated concentrations for 1hr. A dose range of 0.128 nM to 10,000 nM was evaluated. Inhibitor/cytokine complexes were incubated for 30min at RT on their respective plates (IL-17RA:IL-17A, IL-17RB:IL-17E, IL-17RC:IL-17F, IL-17RE:IL-17C). Plates were washed 3X with TBST and incubated for 30min with Streptavidin-HRP (#21130; Pierce). Plates were washed 3X with TBST and the colorimetric signal was developed with TMB (#TMBW-1000-01; BioFX) and the reaction stopped with 450nm liquid stop solution (#LSTP-1000-01; BioFX). OD values were read on a SpectraMax M3 (Molecular Devices). Percent inhibition was calculated by subtracting the mean IL-17 alone OD values and then dividing by the IL-17 alone OD value (subtracted from the basal OD values).

## Results

### Selection of phage libraries against IL-17A

Two peptide libraries each consisting of 18 random amino acids were selected against 100 nM of Avi-tagged IL-17A. After three rounds of selection were completed, the eluted phage pool was deconvoluted by filter-lift [27]. Out of fifty hits that were sequenced, two peptide sequences, 585–1 and 18–1 appeared at highest frequency (S1 Table). Phage bearing the two peptides were amplified and their specific binding to IL-17A was confirmed in a titer dependent ELISA. The next step involved affinity maturation of each peptide. To identify the minimum pharmacophore of the peptides, each peptide was first truncated. This was achieved by eliminating one amino acid at the time from either the N- or C-terminus until the cysteine residues were reached [28]. Binding of truncated peptides (as displayed on phage) to IL-17A was compared to the parental peptide in a titer dependent ELISA to identify the minimum peptide length by which the peptide retained its specific binding to IL-17A (S1 Table). This was followed by alanine scanning of the truncated peptide to define parameters for subsequent construction of affinity maturation libraries. If mutation of a residue to alanine abrogated binding of the peptide to IL-17A, that residue was marked as a hotspot and was not changed. If mutation of a residue to alanine did not lead to a significant change in binding of the peptide, that residue was fully (NNK) randomized [18, 29, 30]. Finally, if mutation of a residue to alanine reduced binding of the peptide at some intermediate degree, then that residue was soft randomized, meaning that the library was designed with a bias towards the parent residue at that position; 50% of the time the parental residue occurred at that position and the remaining 50% all other amino acids appeared (S1 Table). Based on all this information, affinity maturation libraries were constructed for each peptide (S1 Table). To reduce avidity due to presence of multiple copies of peptides on phage, a monovalent display format was used. The libraries were selected under more stringent conditions to favor selection of high-affinity peptides with slow off-rates. Titer dependent binding of the selected peptides to 50 nM Avi-tagged IL-17A was confirmed and select peptides were synthesized as free peptides for further analysis (Table 1).

**Table 1:** Affinity matured peptide characterization.

Peptide	Sequence	SPR			AlphaLISA
		ka1 (1/Ms)	kd1 (1/s)	KD1 (μM)	IC_50_ (μM)
	1 6 10 15 20				
585–1	DSSAVCWAFPHHPLCHMKAT	8.99E+04	0.01315	0.15	4.98 (n = 1)
585–865	ADADMCWFFPTSPWCH----	1.34E+05	0.1227	0.92	0.67 ± 0.62 (n = 2)
585–870	DLSAVCWAFPWDPECH----	2.24E+05	0.00658	0.03	0.016 ± 0.013 (n = 3)
585–876	DSSAVCWAFPYLPECH----	9.33E+04	0.0162	0.17	0.048 ± 0.018 (n = 2)
585–869	DISAVCWAFPFDPECH----	6.45E+04	0.016	0.25	0.039 ± 0.018 (n = 2)
	1 5 10 15				
18–1	AYECPRLEYDMFGALHCLPS	2.29E+03	0.0282	12.30	61.7 (n = 1)
18–927	--CPRLEYDMFGALHCL--	7.04E+03	0.0286	4.06	5.3 ± 2.5 (n = 3)
18–906	---CLDLQYDPWGALHCI--	6.25E+03	0.016	2.56	2.0 ± 1.3 (n = 2)
18–903	---CFDLQYDPWGALHCI--	7.57E+03	0.0183	2.41	1.5 ± 0.4 (n = 2)
18–913	---CLDLQYDMFGALHCV--	1.37E+04	0.0528	3.87	1.9 ± 0.7 (n = 2)
18–921	---CLDLVYDPWGALHCI--	5.51E+03	0.0223	4.04	3.1 ± 1.2 (n = 2)
18–902	---CWVLEYDMFGALHCR--	1.35E+03	0.0175	1.30	1.9 ± 1.5 (n = 3)
18–964	---CWALEYDMFGYLHCR--	3.17E+04	0.00922	0.29	0.72 (n = 1)
18–972	---CWVLEYDMFGFLHCR--	6.31E+04	0.00457	0.072	0.20 (n = 1)
18–967	---CWVLEYDMFGYLHCR--	1.39E+05	0.0102	0.073	0.19 (n = 1)

ND: Not Determined

### SPR analysis of IL-17A binding peptides

**Fig 1:**
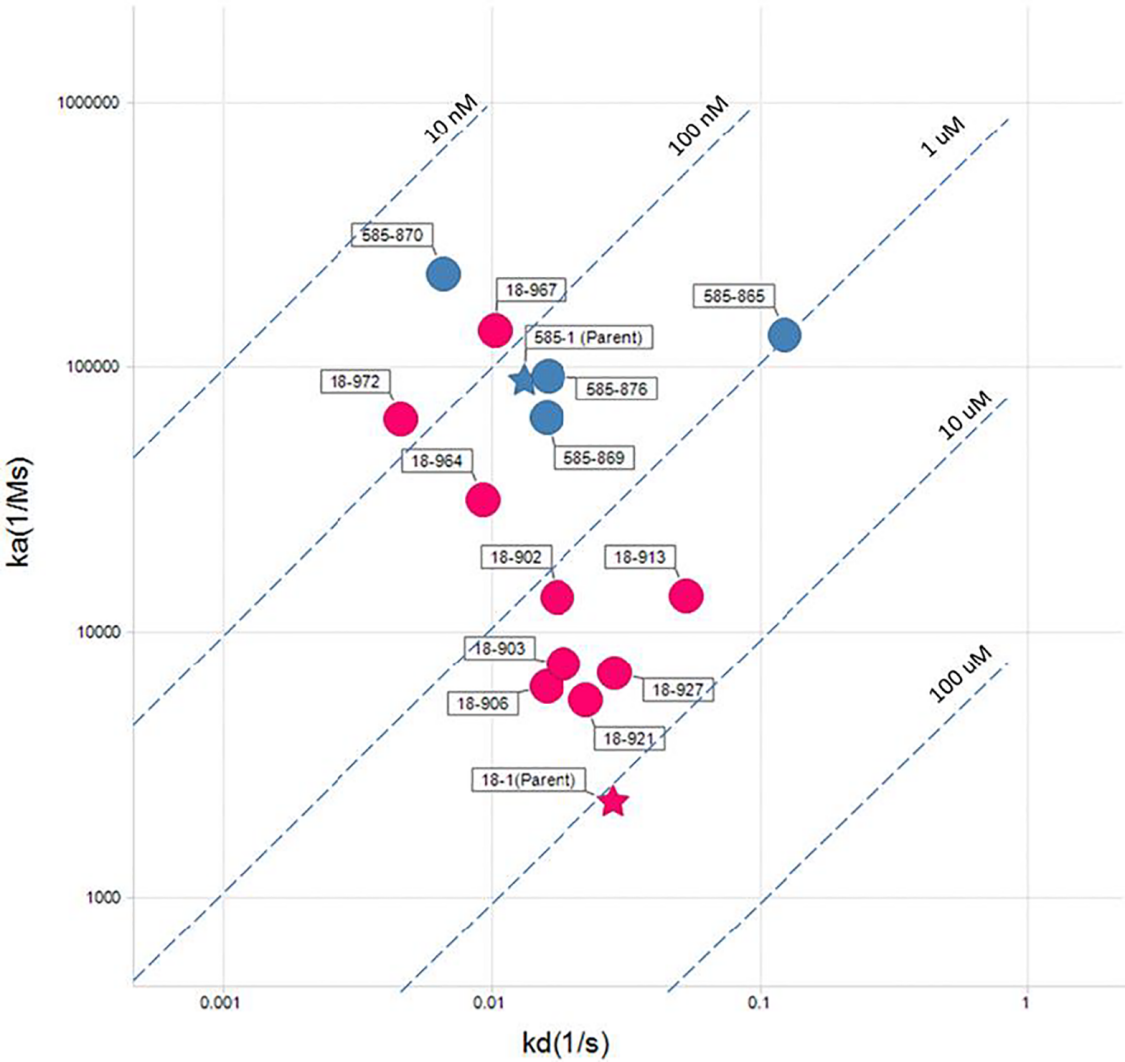
On-& off- rate map of the the two peptide series. 585–1 & 18–1 peptide series are shown in in blue and red, respectively. Parent peptides are shown as stars and the affinity matured offspring as circles. Affinity is indicated by dashed lines.

The binding activity of the resulting free peptides was confirmed using SPR (Table 1 and S1 Fig). The parent peptide 585–1 bound to IL-17A with K_D_ of 146 nM, 84-fold tighter than the parent peptide 18–1 (Table 1). The two parent peptides bound to IL-17A with a comparable k_off_. However, the k_on_ of the 585–1 peptide was 39-fold faster than the 18–1 peptide (8.99e+4 vs 2.29e+3 1/Ms). Affinity maturation of both series produced peptides with higher affinity than their parents (Fig 1) and as a result peptides with 5- and 170-fold higher affinity were identified for 585–1 and 18–1 series, respectively. In contrast to the stronger binding observed when displayed on phage, as free peptides in rare instances, a few affinity matured sequences had lower affinity than their parents (for example 585–865). In those cases, the residues adjacent to the peptide sequence (from phage) may have contributed to overall increase affinity observed when the peptides were displayed on phage.

### HDX analysis

**Fig 2:**
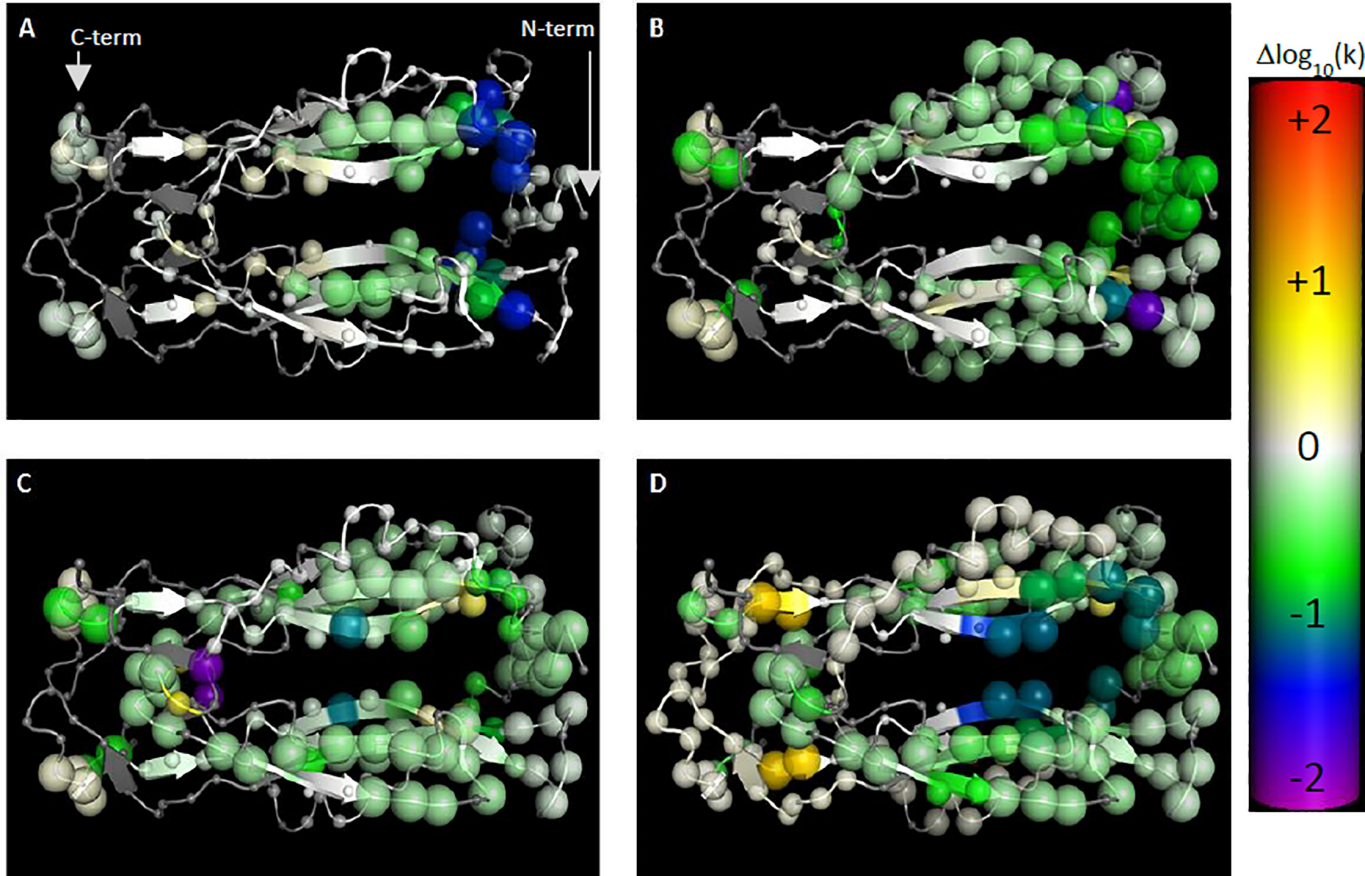
Differential HDX of IL-17A in complex with 4 peptides vs the apo state, illustrated on a model of IL-17A built from 4HSA. The color (generated using the pymol spectrum command) of each alpha carbon represents the magnitude of the change in calculated H-D exchange rate constant upon binding of the corresponding ligand, with purple < = -2.0, blue, green, white = 0.0, yellow, orange, red > = +2.0 log units. The size of each sphere indicates confidence that the apparent signal is truly non-zero, based upon the mean and standard deviation of the acceptable models from the Bayesian analysis, with 0.5 Å radius indicating zero confidence and 1.5 Å indicating a confidence of 1.0 [21]. The blue and green toward the right of the protein upon binding of 585-1-series peptides shows protection to exchange suggestive of binding in that region, while the blue and green closer to the center of the structure in the 18-series peptides suggests binding in that zone. Differential HDX of IL-17A in complex with 585–1 (A), 585–870 (B), 18–1 (C), and 18–902 (D).

The HDX-MS experiment can measure direct and allosteric changes in protein dynamic upon ligand binding. Therefore, we applied HDX to determine the binding epitope of the active phage peptides using the approach described previously [20]. Peptide 585–1 bound to the “alpha helix” pocket region of IL-17A (Figs 2A and S2A), a region that has shown to bind to the alpha helix of IL-17RA [12]. As shown in Fig 2C and S2C Fig, peptide 18–1 bound to a completely distinct region, the larger beta-hairpin pocket, an area that interacts with the beta hairpin region of IL-17RA [12]. Peptide 18–1 extends towards the α-helix region, but it does not enter into the pocket and its binding site remains in the β-hairpin pocket. Since there is crystallographic and chemical protein modification evidence that the Ensemble macrocycle and related ligands bind to the β-hairpin pocket [31, 32] (and this was previously predicted using the same HDX-based methods [20]), the more probable explanation is that the observed HDX effects of the peptides described here are also due to direct binding and what is observed is not the result of peptide induced conformational change in IL-17A. After affinity maturation, peptides derived from each of these starting points were examined by HDX to ensure that their binding epitopes remained the same. As shown in Fig 2A, 2B and S2A and S2B Fig for 585–1 and its affinity matured peptide 585–870, the binding epitope did not change drastically. However it is interesting to note that the protection to exchange afforded by 585–870 is observed generally in a smaller degree but across a larger range of residues than 585–1, including the flexible loop region of IL-17A near the N-terminus. For 18–902 the protection is essentially on the same residues but is stronger than for 18–1 (Fig 2C and 2D, S2C and S2D Fig). This may suggest that the affinity maturation process achieved the observed improvement in SPR affinity in the two series via different means, with the main improvement in 585–1 being derived from a more extended interaction with the protein and the main improvement in 18–1 being derived from stronger interactions with the same protein region.

### Crystal structure of peptide with IL-17A

**Fig 3:**
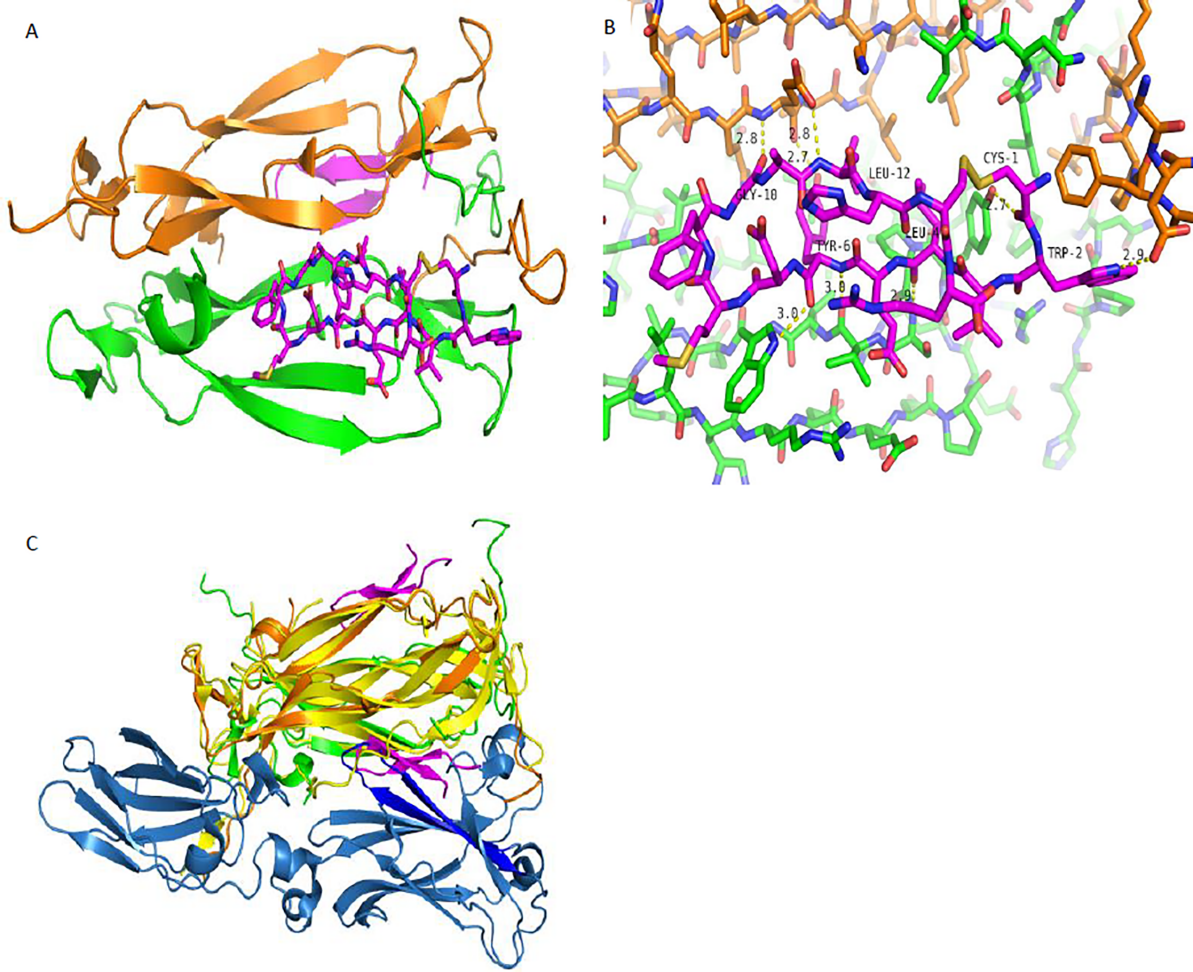
Crystal structure of IL-17A in complex with peptide 18–902. A) The IL-17A dimer binds two peptide molecules, with each peptide molecule occupying one of the two symmetrically equivalent dimer interfaces. IL-17A is depicted in cartoon representation, with one monomer colored in green and the second monomer colored in orange. The peptide molecules are colored magenta, with one of the molecules depicted in stick representation and the other is shown in cartoon representation to reveal the secondary structure. B) Close-up view of the interactions between IL-17A and 18–902. The peptide interacts with IL-17A through both Van Der Waals and hydrogen bonds. The interaction is mediated primarily by peptide main chain atoms interacting with both main chain and side chain atoms of IL-17A. C) Comparison of the IL-17A:18–902 structure with the structure of the IL-17RA:IL-17A complex. Both structures are shown in cartoon representations. In the IL-17:IL-17RA complex, IL-17RA is colored blue and IL-17A dimer is colored yellow. 18–902 adopts a two-stranded anti-parallel β-sheet linked at the ends by a disulfide, that binds in the same pocket as a pair of anti-parallel β-strands (highlighted in bright blue) of IL-17RA.

Despite attempts to co-crystallize peptides from both the 18–1 and 585–1 families, only the 18–1 family yielded usable crystals. In particular, the structure of IL-17A in complex with peptide 18–902 is crystallized in space group P2_1_2_1_2 with two “half-molecules” in the asymmetric unit. The two biological dimers of IL-17A are generated by the crystallographic two-fold axis. As a result, the individual monomers in each of the two IL-17A dimers are related by perfect two-fold symmetry. Each IL-17A dimer binds a pair of peptides that sit at the dimer interface on opposite sides of the dimer (Fig 3A). The diffraction resolution of 1.6 Å yielded a well-defined structure that refined to an R-work of 18.5% and an R-free of 21.2%, with 100% of the residues in the allowed regions of the Ramachandran plot and 98.2% of residues in the favored regions (S2 Table). All fifteen residues of the peptide are clearly ordered and were modeled in both copies. The two independent IL-17A polypeptides in the crystallographic asymmetric unit are very similar, except for C-terminal residues 128–131 that are ordered in one copy but not in the other. In both copies, the internal IL-17A loop (Arg31-Ser40), which has been observed to be ordered in the complex with receptor [12], is disordered in the peptide complex. The 84 overlapping residues in the two copies of IL-17A superimpose with an r.m.s.d. of 0.2 Å. The conformation and interactions of IL-17A with the peptide are identical in the two copies. The bound peptide forms a β-hairpin structure in the complex, which is covalently linked at the ends by the disulfide bond between Cys1 and Cys14 and held together by antiparallel β-sheet hydrogen bonds between Val3, Glu5, Asp7 and Arg15, His13, Ala11, respectively. The interactions between the peptide and cytokine are dominated by hydrogen bonds by main chain atoms (Fig 3B) with the carbonyl of Cys1 interacting with the hydroxyl of A:Tyr62; the carbonyl of Leu4 interacting with the main chain amino group of A:Ser64; the amino nitrogen and the carbonyl of Tyr6 interacting with the main chain carbonyl of A:Val65 and the sidechain of Trp67 respectively; and the carbonyl of Gly10 and the amino nitrogen of Leu12 hydrogen bonding to the amino nitrogen and carboxylate sidechain of B:Glu95 respectively. The side chain of Tyr6 inserts into a pocket at the dimer interface and makes a hydrogen bond to the main chain carbonyl of B:Glu95, while the side chain of Met8 packs between the cytokine side chains of Tyr44, Leu53, and Trp67. A comparison of the published structure of the IL-17A/IL-17RA complex reveals that pocket occupied by the peptide β-hairpin overlaps with a β-hairpin of the receptor in its interaction with the cytokine (Fig 3C).

### Determining the peptide activity by AlphaLISA

Pure peptides from the 585–1 and 18–1 families were assayed in AlphaLISA format to investigate the potential for disruption of the IL-17A/IL-17RA cytokine-receptor interaction in a biochemical format. In general, the IC_50_ values determined by AlphaLISA agreed quite closely with the K_D_ values determined through SPR analysis (Table 1). The 585–1 family of peptides demonstrated a 7 to 312-fold increase in potency upon affinity maturation with 585–870 demonstrating the highest inhibitory activity of 16 nM (Table 1). The 18–1 family of peptides similarly showed a 12 to 30-fold increase in potency upon optimization, albeit to a lesser degree than the 585–1 family (Table 1). As was observed in SPR for this family, the differences in AlphaLISA activity between the affinity matured peptides were not significant, with several peptides having IC_50_’s in the 0.2 to 2.0 μM range.

### Determining the peptide activity by a functional cell based assay

**Fig 4:**
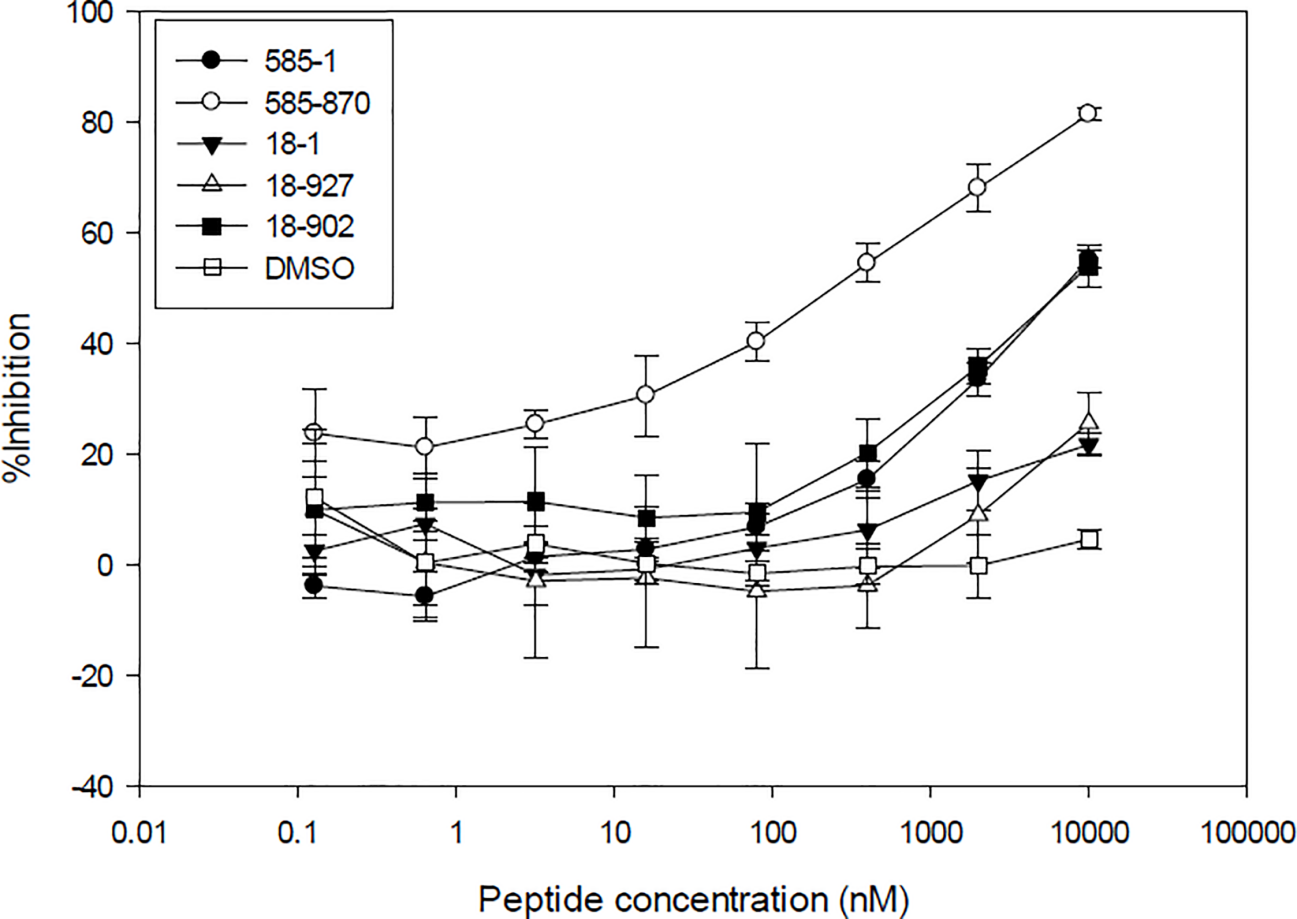
Peptide neutralization of human IL-17A in a cell-based assay. Peptides were incubated at the indicated concentrations with IL-17A prior to addition to HT-29 cells. After 48 hr. incubation, the culture supernatant was collected and inhibition of CXCL1 production was determined by ELISA. Results are shown as the mean of duplicate treatments ± SD and are representative of two independent experiments.

The human colorectal adenocarcinoma epithelial cell line, HT-29, was utilized previously to show antibody neutralization of human IL-17A. The efficacy of antibodies was measured by determining the inhibition of CXCL1 (GROα) expression [7]. A similar approach was used here to screen peptides for the ability to neutralize the biological activity of human IL-17A. Peptide 18–1 showed minimal inhibition of human IL-17A-induced CXCL1 expression even at the highest concentration tested (Fig 4). After affinity maturation, 18–902 demonstrated a measurable inhibition of CXCL1 production with 38.4% inhibition at 10 μM. Peptide 585–1 showed a dose-dependent inhibition of IL-17A-induced CXCL1 production by the HT-29 cells with 42.5% inhibition at 10 μM (Fig 4). The dose-dependent inhibition was significantly increased with the affinity-matured peptide, 585–870 with 76.6% inhibition at 10 μM. No inhibition was observed with the DMSO vehicle alone.

### ELISA-based specificity assay

**Fig 5:**
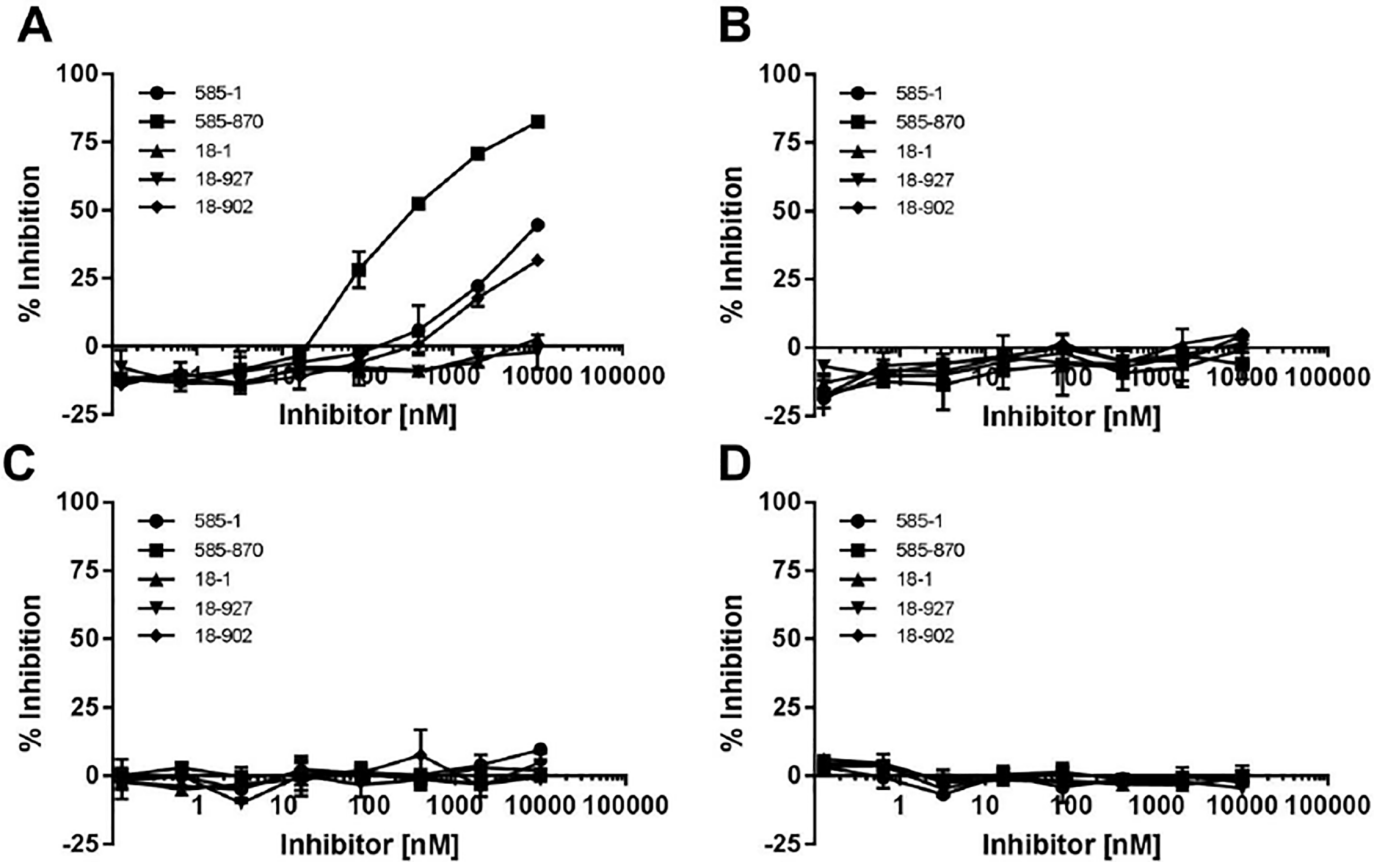
Specificity of peptide neutralization on IL-17 family members. ELISA plates were coated O/N with (A) IL-17RA-Fc, (B) IL-17RE-Fc, (C) IL-17RB-Fc, or (D) IL-17RC-Fc. Indicated concentrations of peptides were pre-incubated with biotinylated (A) IL-17A, (B) IL-17C, (C) IL-17E, or (D) IL-17F for 1 h. Peptide:cytokine mixtures were then added to the respective ELISA plate for 30 min prior to developing. Results are shown as the mean of duplicate treatments ± SD and are representative of two independent experiments.

An ELISA-based biochemical assay was utilized to determine selectivity of the peptides to inhibit human IL-17A and other IL-17 family members. As was observed in the cell-based IL-17A neutralization assay, peptide 18–1 showed negligible inhibition (2.9% at 10 μM), whereas the affinity matured 18–902 demonstrated 32% improved inhibition of human IL-17A binding to IL-17RA (Fig 5A). The peptide 18–927 demonstrated no inhibition. Peptide 585 showed 44.6% inhibition at 10 μM and after affinity-maturation this was enhanced to 82.5% with 585–870. The specificity to other IL-17 family members was determined by measuring the ability of the peptides to inhibit the interaction of the cytokines to their receptor. None of the peptides tested inhibited the binding of IL-17C to IL-17RE (Fig 5B), IL-17E to IL-17RB (Fig 5C), or IL-17F to IL-17RC (Fig 5D). The results indicate the peptides are highly selective in inhibiting human IL-17A.

## Discussion

IL-17A belongs to a family of proteins that constitute protein-protein interaction (PPI) targets. Like other members of the PPI family, the interface between IL-17A and its receptor is broad, flat, and largely devoid of grooves that are suitable for binding traditional small molecules. Instead, it contains single, isolated residues that contribute to most of binding free energy in the interface [10, 11, 14]. These residues are called “hot spots” and usually cover less than 10% of the entire interface [14]. Interestingly, hot spots are not randomly distributed along the surface. They tend to form clusters near the center of the interface to form “hot regions”. These hot regions are known to be druggable and dynamic to facilitate binding of the protein to a set of structurally diverse partners, including peptides and small molecules [11]. Two such hot regions were previously identified for IL-17A through close inspection of its structure in complex with IL-17RA. These two regions, called α-helix and β-hairpin pockets are named based on the structural determinants of IL-17RA that interact with the cytokine [20]. The α-helix (residues 60–62, 99, 101, 110) and β-hairpin (residues 93–95) pockets (S3 Fig) reside within the previously defined regions I and II, respectively [12, 20]. Both pockets are considered viable options for binding of small molecules and macrocycles. In the present study, we utilized combinatorial peptide phage display, HDX, crystallography, and functional assays to map the IL-17A interface with IL-17RA to gain more insight into the specificities of this interaction. As a result, we identified two novel peptides that bind to IL-17A at two hot regions: α-helix and β-hairpin pockets.

HDX analysis of the two parental peptides, 585–1 and 18–1, suggested that they bind to α-helix and β-hairpin pockets of IL-17A, respectively. Affinity maturation of both peptides resulted in the discovery of stronger binding peptides when compared to the parental peptides (Table 1). The affinity matured peptides interact with IL-17A on the same epitope as the parental peptides (Fig 2). After affinity maturation, the 18–902 and 585–870 showed enhanced inhibition compared to the respective parent peptides in both biochemical and cell-based assays (Fig 4, Table 1). The inhibition observed with the different peptides was comparable between the two assays. Additionally, the parental and affinity matured peptides were highly selective for the inhibition of human IL-17A and did not demonstrate activity with the three other human IL-17 family members tested (Fig 5). This result was not surprising since IL-17A shares a low sequence identity with its other family members in both α-helix and β-hairpin regions [5, 12].

Without a crystal structure to provide detail on the 585 series binding mode and the much smaller dynamic range of the effect of substitution on affinity, it is difficult to be certain as to why some changes are tolerated and others are not. Perhaps the most significant change is the introduction of the larger Trp at position 11 in 585–870 as compared to the His11 in the parent 585–1 (Table 1). This might be the main contributing factor in the 5-fold increase in affinity. This can be explained by the increase in the extent of the interaction between the peptide and IL-17A, which agrees with the more extensive protection pattern observed in HDX.

**Fig 6:**
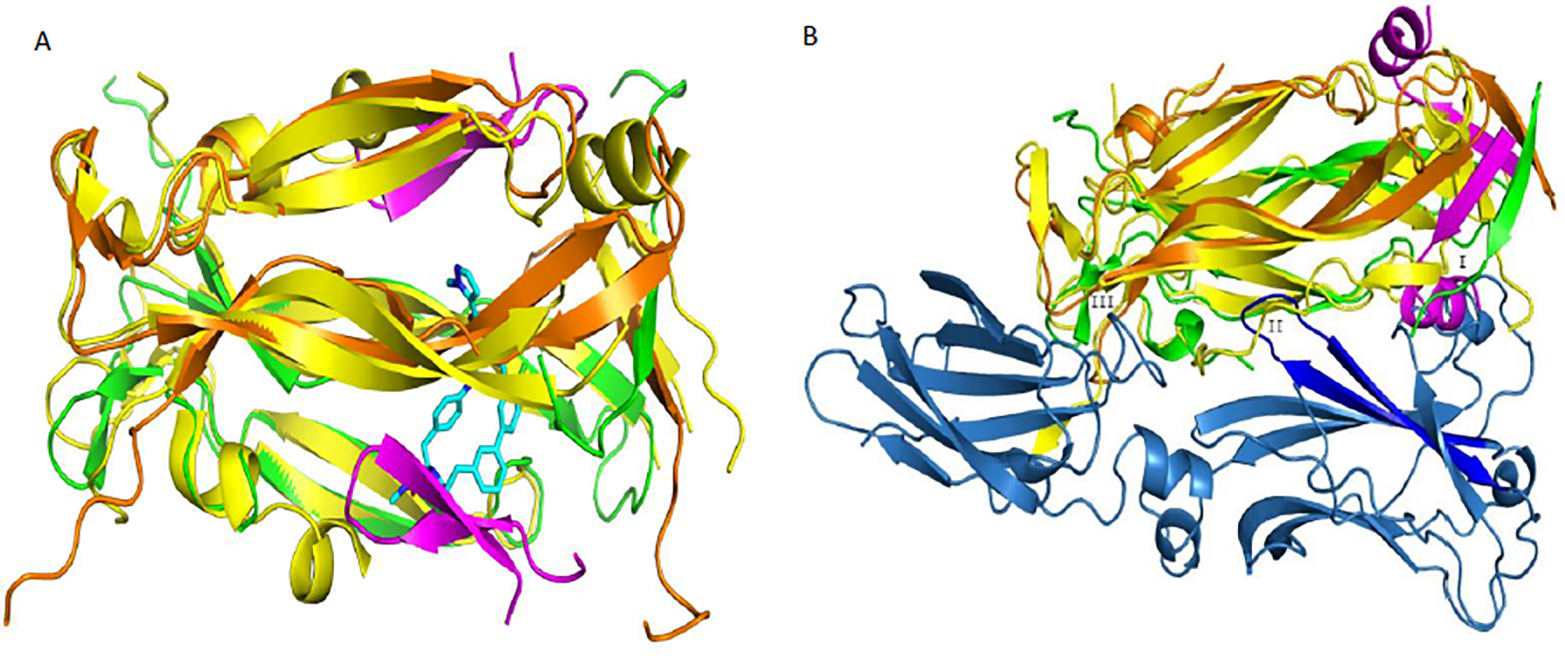
Comparison of the crystal structure of IL-17A in complex with peptide 18–902 to the Pfizer macrocyclic ligand and peptide. A) Comparison of the structure of 18–902 with Pfizer macrocyclic ligand [32]. In the peptide complex, the IL17A monomers are colored green and orange and the peptide molecules are colored magenta. Pfizer macrocyclic is depicted in cyan in stick representation. B) Comparison of the structure of the linear peptide in complex with IL-17A [31], with the structure of the IL17RA:IL17A complex using the same color scheme as Fig 3C. The linear peptide is depicted in magenta cartoon representation.

The crystal structure of the IL-17A complex with peptide 18–902 confirmed the HDX result that 18–902 binds to β-hairpin region of IL-17A. Similar to IL-17RA, peptide 18–902 assumes a β-hairpin conformation in the context of β-hairpin pocket of IL-17A (Fig 3C). The structure also provides a structure-based rationale for the observed inhibition of IL-17RA binding to the cytokine in the presence of peptide. Comparison of our structure to the published structure of the IL-17A/IL-17RA complex reveals that the IL-17A pocket occupied by the 18–902 overlaps with the β-hairpin (between strands C-C’) of the receptor while interacting with the cytokine (Fig 3C) [12]. Therefore binding of 18–902 on both sides of the IL-17A dimer should structurally obstruct binding of the receptor. In addition to 18–902 and IL-17RA, Ensemble macrocycles [20] and Pfizer’s small molecule compounds bind to the β-hairpin pocket (Fig 6A) [32], suggesting that this pocket is highly druggable. The Pfizer ligand binds at the middle of the IL-17A dimer, pushing apart the two monomers, and extending into the β-hairpin pocket, while peptide 18–902 binds in the β-hairpin pocket at the surface of the dimer to obstruct receptor binding (Fig 6A). Binding of Ensemble and Pfizer macrocyclic ligand antagonists to the induced pocket at the center of the IL-17A dimer (close to region II) have been shown to cause conformational changes that also prevent IL-17RA binding [20, 32].

In addition, the crystal structure of the IL-17A complex with 18–902 provides insights into how the affinity maturation of the parental 18–1 resulted in an increase in its binding affinity. One notable modification is the change of residue 2 (affinity matured peptide numbering) from Pro to Leu, Phe, or Trp. The change to a non-cyclic but still hydrophobic amino acid at this position may have reduced strain in the bound vs. the free form, leading to modest improvement in affinity. Trp in particular is able to form the hydrogen bond observed in the crystal structure to the sidechain of Asp15 of the cytokine (Fig 3B), which may explain the higher affinity of peptides 18–902, 18–964, 18–972, and 18–967 (Table 1). Residues 3 and 15 are spatially close to each other with a Cα separation of 5.2 Å. These two residues are located on the solvent-exposed face of IL-17A next to multiple polar residues, including acidic and basic residues. It appears that one of these residues is required to be hydrophobic while the other must be charged. Interestingly, either residue can fulfil either role in order to fit into the pocket. The residue at position 5 is generally Gln or Glu, consistent with its location on the solvent exposed surface of the peptide and proximity to Arg55 of the cytokine. Finally, the two residues at positions 8 and 9 are either Met-Phe or Pro-Trp. In the crystal structure, the Met-Phe combination can be seen to fill a hydrophobic subpocket on the cytokine surface formed by Tyr44, Leu53 and Trp67 sidechains. Replacing the Met with Pro appears to be perfectly feasible since the N-H of the Met residue is solvent exposed and the orientation is appropriate. However, this may leave a portion of that subpocket unoccupied unless a complementary change is made. Then the preferred combination of Pro-Trp might fill unoccupied part of the pocket with the larger sidechain of the Trp. Thus, all of the changes are consistent with a binding mode similar to that observed in the crystal structure of 18–902. This also appears reasonable on the basis of the information obtained by HDX.

Comparison of 18–902 peptide complex to the recently published structure of IL-17A in the presence of a linear peptide, called HAP [31] reveals that the two peptides adopt different conformations and bind to different regions of the cytokine (Fig 6B). As mentioned previously, 18–902 adopts a β-hairpin conformation and binds to the middle of the IL-17A dimer. In contrast, the linear peptide binds to the N-terminal end of the cytokine dimer and inserts a β-strand between β-strand 4 of one IL-17A monomer and β-strand 0 of the second monomer, followed by a short C-terminal helix that interacts with the second IL-17A monomer (Fig 6B). The two peptides prevent receptor binding by disrupting different regions of the extended IL-17RA/IL17A surface. The linear peptide described by Liu *et al*. intercalates at the so called “Region I” of the IL-17A/IL-17RA interface. Peptide 18–902 disrupts Region II of the interface but does not perturb the structure of the cytokine. Binding site of 585 peptide series is also distinct from binding site of HAP. N-terminal region of IL-17A shows increased protection with 585–870 (Fig 2B), but the HAP peptide severely disrupts that region of the dimer. While there is no significant change in the beta sheet protection with 585–870, the beta sheet secondary structure is significantly disrupted in the structure of HAP in complex with IL-17A [31].

It is also important to note that unlike the structure of the IL-17A complex with 18–902 presented here, the structure of the linear peptide by Liu *et al*. in complex with IL-17A could only be obtained in ternary complex with an anti-IL-17A Fab. Similarly, the macrocyclic small molecule inhibitor complex with IL-17A reported by Pfizer could only be obtained as a quaternary complex with the anti-IL-17A Fab and the linear peptide.

In conclusion, we have discovered two novel peptides that bind to druggable pockets of IL-17A. Epitope determination as well as physical and biochemical characterization of both peptides confirmed druggability of these two distinct pockets on IL-17A. The 585 and 18 peptide series bind to IL-17A independently in a non-competitive manner (S4 Fig). This suggests that a synergistic inhibition of IL-17/IL-17R may be achieved if these peptides were used in combination. Additionally, these peptides can be used as valuable tools for discovery and characterization of specific small molecule IL-17 antagonists.

## Supporting information

S1 Fig **SPR sensorgrams of the two parent peptides 585–1 (A) & 18–1 (B) and their respective best affinity matured offsprings 585–870 (C) and 18–972 (D).** Y-axis represent SPR response level in RU and X-axis represent time in second. Curve fitting were performed globally using a 1:1 binding model (black lines). The highest dosing concentration of the peptides were 2 μM for 585, 30 μM for 18, 1 μM for 585–870, and 3 μM for 18–972.
(DOCX)

S2 Fig Deuterium uptake plots of peptides 585–1, 585–870, 18–1, and 18–902. A & B) Deuterium uptake plots of peptide 585–1 and 585–870 against IL-17A segments 102–116 and 103–114 originating in the α-helix pocket, respectively. C & D) Deuterium uptake plots of peptides 18–1 and 18–902 against IL-17A segments 90–101 and 92–99 originating in the β-hairpin pocket.
(DOCX)

S3 Fig HDX peptide map of IL-17A. Numbering starts at the first IL-17A residue. Bracketed regions indicate peptide coverage by MS.
(DOCX)

S4 Fig Additive binding of 585–870 and 18–902 suggested non-competitive binding mode of the two peptides. Simulation using BiaSilumation software, GE Healthcare, of binding of hypothetical peptide 1 (MW = 1875, ka = 1e5 1/Ms, kd = 5e-2 1/s) at saturating concentration of 10 μM and hypothetical peptide 2 (MW = 1875, ka = 1e4 1/Ms, kd = 1e-2 1/s) at 1 μM are shown in A-red solid & A-blue solid respectively; simulation of same peptide 1 & 2 in mixture in competitive and non-competitive mode are shown in A-black dash & A-green dash, respectively. Experimental binding sensorgrams of 10 μM 585–870 and 0.3 μM of 18–902 are shown in B-red & B-blue, respectively. Experimental sensorgram of the mixture of 10 μM of 585–870 & 0.3 μM of 18–902 is shown in B-green. The sensorgram of the mixture showed additive effect of both peptides and matched the non-competitive profile in simulation.
(DOCX)

S1 Table Phage library and peptide characterizations. (DOCX)

S2 Table Data collection and refinement statistics. (DOCX)
