# Supplementary material for: Utilization of peptide phage display to investigate hotspots on IL-17A and what it means for drug discovery
Source: PLoS One. 2018 Jan 12;13(1):e0190850. doi: 10.1371/journal.pone.0190850 (PMC5766103; doi:10.1371/journal.pone.0190850)

**Supporting information**

**S1 Fig**. **SPR sensorgrams of the two parent peptides 585-1 (A) & 18-1 (B) and their respective best affinity matured offsprings 585-870 (C) and 18-972 (D).** Y-axis represent SPR response level in RU and X-axis represent time in second. Curve fitting were performed globally using a 1:1 binding model (black lines). The highest dosing concentration of the peptides were 2 µM for 585, 30 µM for 18, 1 µM for 585-870, and 3 µM for 18-972.


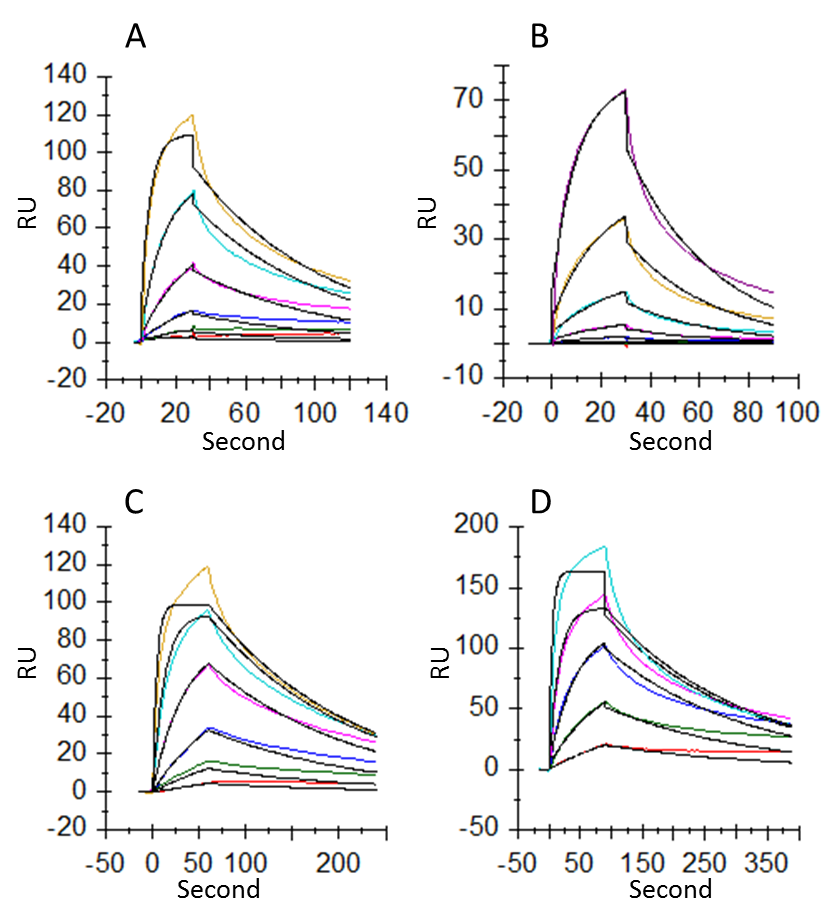

Supplement: S1 Fig — SPR sensorgrams of the two parent peptides 585–1 (A) & 18–1 (B) and their respective best affinity matured offsprings 585–870 (C) and 18–972 (D). Y-axis represent SPR response level in RU and X-axis represent time in second. Curve fitting were performed globally using a 1:1 binding model (black lines). The highest dosing concentration of the peptides were 2 μM for 585, 30 μM for 18, 1 μM for 585–870, and 3 μM for 18–972. (DOCX) [file pone.0190850.s001.docx]
