# Supplementary material for: Utilization of peptide phage display to investigate hotspots on IL-17A and what it means for drug discovery
Source: PLoS One. 2018 Jan 12;13(1):e0190850. doi: 10.1371/journal.pone.0190850 (PMC5766103; doi:10.1371/journal.pone.0190850)
Supplement: S2 Fig — A & B) Deuterium uptake plots of peptide 585–1 and 585–870 against IL-17A segments 102–116 and 103–114 originating in the α-helix pocket, respectively. C & D) Deuterium uptake plots of peptides 18–1 and 18–902 against IL-17A segments 90–101 and 92–99 originating in the β-hairpin pocket. (DOCX) [file pone.0190850.s002.docx]

**Supporting information**

**S2 Fig. Deuterium uptake plots of peptides 585-1, 585-870, 18-1, and 18-902.** A & B) Deuterium uptake plots of peptide 585-1 and 585-870 against IL-17A segments 102-116 and 103-114 originating in the α-helix pocket, respectively. C & D) Deuterium uptake plots of peptides 18-1 and 18-902 against IL-17A segments 90-101 and 92-99 originating in the β-hairpin pocket.

**A)**

**B)**

**IL-17A+585-1**

**IL-17A**

**IL-17A**

**IL-17A+585-870**

**102-116 (+4)**

**102-116 (+4)**

**α-Helix Pocket**

**103-114 (+3)**

**103-114 (+3)**

**D)**

**C)**

**IL-17A**

**IL-17A+18-1**

**90-101 (+2)**

**92-99 (+2)**

**90-101 (+2)**

**92-99 (+2)**

**IL-17A**

**IL-17A+18-902**

**β-Hairpin Pocket**
