# Supplementary material for: Utilization of peptide phage display to investigate hotspots on IL-17A and what it means for drug discovery
Source: PLoS One. 2018 Jan 12;13(1):e0190850. doi: 10.1371/journal.pone.0190850 (PMC5766103; doi:10.1371/journal.pone.0190850)
Supplement: S3 Fig — Numbering starts at the first IL-17A residue. Bracketed regions indicate peptide coverage by MS. (DOCX) [file pone.0190850.s003.docx]

**Supporting information**

**S3 Fig**. **HDX peptide map of IL-17A.** Numbering starts at the first IL-17A residue. Bracketed regions indicate peptide coverage by MS.


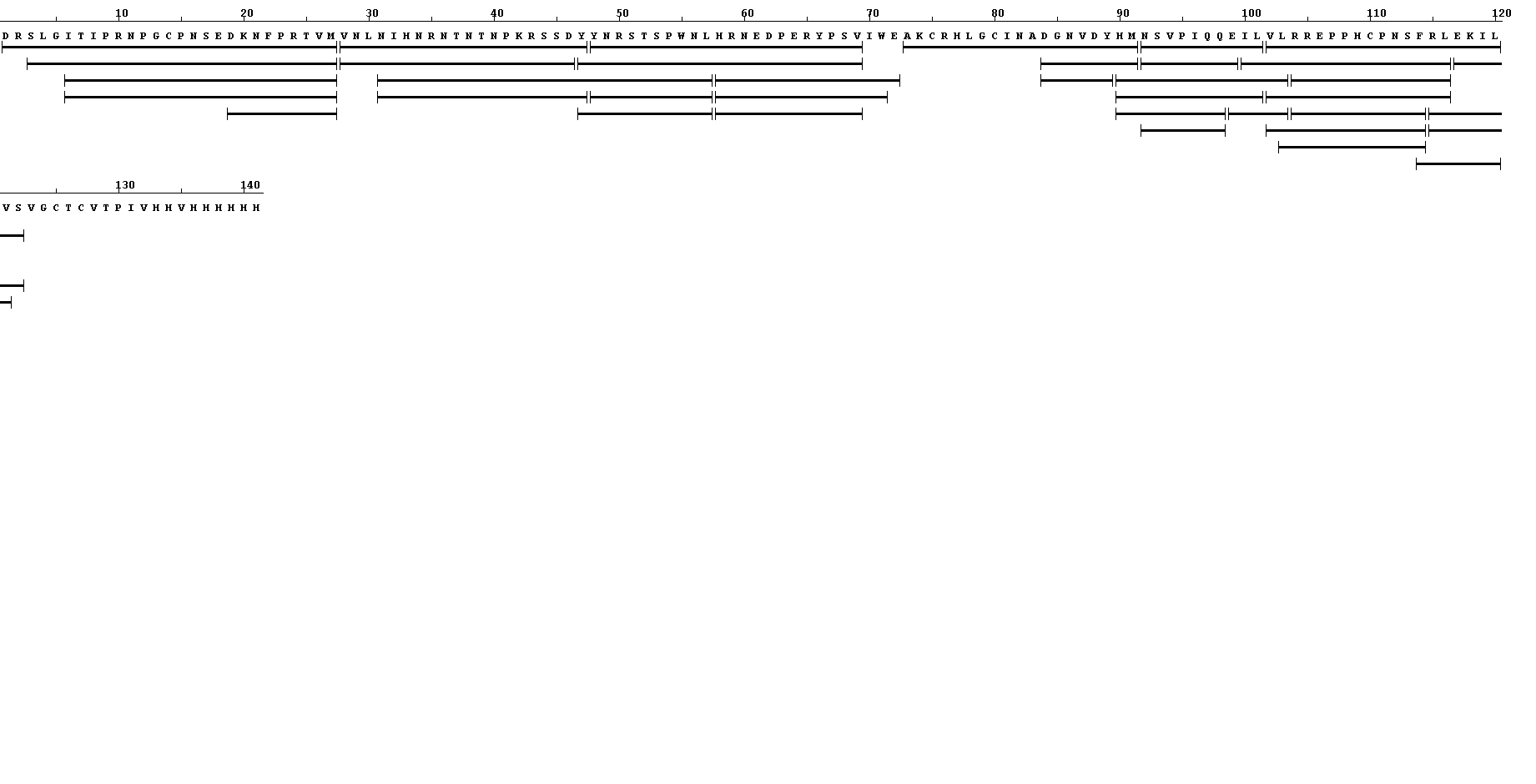


“β-hairpin” pocket

“α-helix” pocket
