# Supplementary material for: Utilization of peptide phage display to investigate hotspots on IL-17A and what it means for drug discovery
Source: PLoS One. 2018 Jan 12;13(1):e0190850. doi: 10.1371/journal.pone.0190850 (PMC5766103; doi:10.1371/journal.pone.0190850)
Supplement: S4 Fig — Simulation using BiaSilumation software, GE Healthcare, of binding of hypothetical peptide 1 (MW = 1875, ka = 1e5 1/Ms, kd = 5e-2 1/s) at saturating concentration of 10 μM and hypothetical peptide 2 (MW = 1875, ka = 1e4 1/Ms, kd = 1e-2 1/s) at 1 μM are shown in A-red solid & A-blue solid respectively; simulation of same peptide 1 & 2 in mixture in competitive and non-competitive mode are shown in A-black dash & A-green dash, respectively. Experimental binding sensorgrams of 10 μM 585–870 and 0.3 μM of 18–902 are shown in B-red & B-blue, respectively. Experimental sensorgram of the mixture of 10 μM of 585–870 & 0.3 μM of 18–902 is shown in B-green. The sensorgram of the mixture showed additive effect of both peptides and matched the non-competitive profile in simulation. (DOCX) [file pone.0190850.s004.docx]

**Supporting information**

To see if the two peptides, 585-870 and 18-902, can simultaneously bind to IL-17A, 10 µM of 585-870 (saturating concentration), 0.3 µM of 18-902, and mixture of 10 µM of 585-870 with 0.3 µM of 18-902 were injected across an IL-17A sensor sequentially. SPR method was the same as described previously except that the assay temperature in this experiment was set at 25°C.

SPR detects density change on the protein sensor as a function of ligand binding and size of the ligand. In a 1:1 interaction system, the response level reaches it maximum (Rmax) when all the ligand pockets on a given protein sensor is occupied by its ligand, also known as saturated. At this point, additional exposure to the same ligand or higher concentration of the ligand would not increase the SPR response level any further. The competition assay described here was designed based on that principle. When peptide is injected across an IL-17A surface at a saturating concentration, addition of a second competitive peptide with the same molecular weight will not change the maximum binding level since the total binding site remains the same, whereas a non-competitive peptide will simply binds to its independent site. As a result, the response level will be additive to those of the 1^st^ peptide. When 18-902 was mixed with saturating concentration of 585-870 and inject across IL17A sensor, it showed an additive binding, which suggested a non-competitive binding mode between the two peptides. This confirmed the HDX result that the two peptides 585-870 and 18-902 bind to two independent sites; α-helical and β-hairpin pockets on IL-17A, respectively.

**S4 Fig. Additive binding of 585-870 and 18-902 suggested non-competitive binding mode of the two peptides**. Simulation using BiaSilumation software, GE Healthcare, of binding of hypothetical peptide 1 (MW=1875, ka = 1e5 1/Ms, kd=5e-2 1/s) at saturating concentration of 10 µM and hypothetical peptide 2 (MW=1875, ka=1e4 1/Ms, kd=1e-2 1/s) at 1µM are shown in A-red solid & A-blue solid respectively; simulation of same peptide 1 & 2 in mixture in competitive and non-competitive mode are shown in A-black dash & A-green dash, respectively. Experimental binding sensorgrams of 10 µM 585-870 and 0.3 µM of 18-902 are shown in B-red & B-blue, respectively. Experimental sensorgram of the mixture of 10 µM of 585-870 & 0.3 µM of 18-902 is shown in B-green. The sensorgram of the mixture showed additive effect of both peptides and matched the non-competitive profile in simulation.

| **A)** | **B)** |
| --- | --- |
| 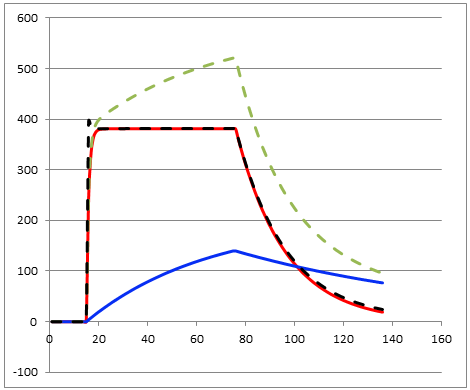 | 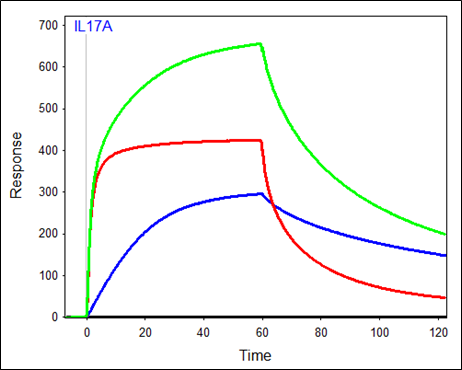 |
