# Supplementary material for: Utilization of peptide phage display to investigate hotspots on IL-17A and what it means for drug discovery
Source: PLoS One. 2018 Jan 12;13(1):e0190850. doi: 10.1371/journal.pone.0190850 (PMC5766103; doi:10.1371/journal.pone.0190850)
Supplement: S1 Table — (DOCX) [file pone.0190850.s005.docx]

**Supporting information**

**S1 Table. Phage library and peptide characterizations**

|  |  | **Diversity** |
| --- | --- | --- |
| 585 library | X_5_CX_8_CX_5_ | 1.4 X 10^10^ |
| 585-1 peptide | DSSAVCWAFPHHPLCHMKAT |  |
| 585-1.T peptide | DSSAVCWAFPHHPLCH---- |  |
| 585-1 (Ala-scan) | DSSAVCWAFPHHPLCH---- |  |
| 585-1 AM library |  | 5.6 X 10^9^ |
| 18-mer library | X_3_CX_12_CX_3_ | 3.7 X 10^10^ |
| 18-1 | AYECPRLEYDMFGALHCLPS |  |
| 18-1.T | ---CPRLEYDMFGALHCL-- |  |
| 18-1 (Ala-scan) | ---CPRLEYDMFGALHCL-- |  |
| 618-1 AM library |  | 3.5 X 10^8^ |

- X is any random amino acid
- Major decrease in binding to IL-17A: hotspot
- Intermediate decrease in binding to IL-17A: soft Randomization
- Minor decrease in binding to IL-17A: NNK Randomization
