# Supplementary material for: Utilization of peptide phage display to investigate hotspots on IL-17A and what it means for drug discovery
Source: PLoS One. 2018 Jan 12;13(1):e0190850. doi: 10.1371/journal.pone.0190850 (PMC5766103; doi:10.1371/journal.pone.0190850)
Supplement: S2 Table — (DOCX) [file pone.0190850.s006.docx]

**Supporting information**

**S2 Table. Data collection and refinement statistics**

|  | Native |
| --- | --- |
| **Data collection** |  |
| Space group | P2_1_2_1_2 |
| Cell dimensions |  |
| *a*, *b*, *c* (Å) | 36.77, 55.12, 144 |
| α, β, γ (°) | 90, 90, 90 |
| Resolution (Å) | 1.70 (1.70-1.79) |
| *R*_merge_ | 0.08 (0.51) |
| *I* / σ*I* | 12.6 (3.0) |
| Completeness (%) | 99.1 (98.3) |
| Redundancy | 6.7 (6.3) |
|  |  |
| **Refinement** |  |
| Resolution (Å) | 1.7 |
| No. reflections | 32662 |
| *R*_work_ / *R*_free_ | 0.182/0.211 |
| No. atoms |  |
| IL-17A | 1716 |
| Peptide | 256 |
| Water | 282 |
| *B*-factors |  |
| IL-17A | 37.8 |
| Peptide | 35.5 |
| Water | 46.6 |
| R.m.s deviations |  |
| Bond lengths (Å) | 0.010 |
| Bond angles (°) | 0.99 |
